# Perforating eyelid injury extending to the brain stem in a 17-year-old woman: a case report

**DOI:** 10.1186/1752-1947-4-18

**Published:** 2010-01-21

**Authors:** Eiichiro Noda, Makoto Inoue, Izumi Yoshikawa-Kobayashi, Toshiyuki Nagamoto

**Affiliations:** 1 Kyorin Eye Center, Kyorin University School of Medicine, Tokyo 181-8611, Japan

## Abstract

**Introduction:**

This case report describes a patient who had a perforating eyelid injury that extended to the brain stem.

**Case presentation:**

A 17-year-old Japanese woman complained of decreased vision in her right eye, with severe ocular pain and headaches, after the metal tip of an umbrella struck her upper right eyelid accidentally. Her vision in the right eye decreased to light perception with commotio retinae, intraretinal hemorrhage, and severe lid swelling. Magnetic resonance imaging (MRI) demonstrated edema of the head of the caudate nucleus and putamen, and the edema extended to the hypothalamus. The MRI findings indicated that the umbrella tip had penetrated through the eyelid and the posterior orbital wall. Vision improved to 20/50 in the right eye, with subretinal fibrosis caused by the choroidal rupture.

**Conclusions:**

We recommend that MRI be performed on the orbit and brain in patients who appear to have symptoms that are inconsistent with the observed injury and when a severe orbitocranial injury is suspected.

## Introduction

Penetrating periorbital wounds are not uncommon, but those that extend to the brain stem are extremely rare [1,2]. Despite the severity of the superficial trauma, injuries that extend into the brain cavity have often been overlooked because they cause only mild symptoms [3]. However, a perforating brain injury is particularly dangerous because a cerebrospinal fluid fistula can lead to meningitis and brain abscess [4]. We describe a patient who suffered accidental perforation of her upper eyelid with the metal tip of an umbrella, and in whom extension of the injury to the midbrain was only identified with computed tomography (CT) and magnetic resonance imaging (MRI).

## Case presentation

**Figure 1:**
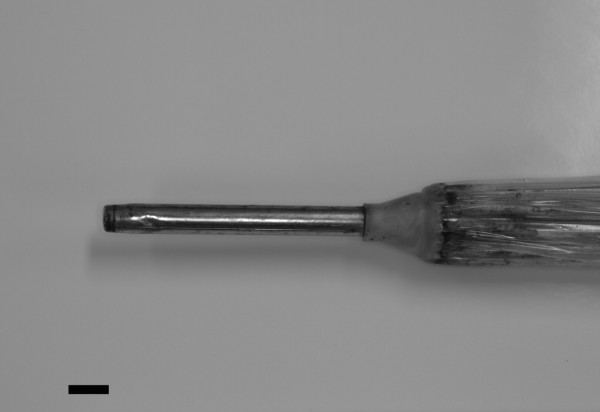
**Photograph of the tip of the umbrella**. The metal tip of umbrella is approximate 70 mm in length and 8 mm in diameter (bar = 10 mm).

A 17-year-old Japanese woman, who had decreased vision in her right eye and severe ocular pain and headaches, was referred for management of vitreous hemorrhage. On a day prior to her visit the woman's younger brother had been playing with an umbrella, which subsequently caused the handle to detach and the metal tip of the umbrella (figure 1) to strike the woman in her upper right eyelid. She reported that she only felt being hit with something hard on her right upper eyelid and the left eye. Following the incident, the umbrella was found on the floor. She visited a local clinic, and the eyelid injury was sutured on the same day because it was considered to be a surface injury. On the next day she had severe ocular pain, headaches, and blurred vision in the right eye and she visited our clinic.

At presentation the lid wound in her right eye was sutured and the lids were severely swollen. Her vision had diminished to light perception only in her right eye and was 20/200 in the left eye, but she could barely open her right eye because of the severe ocular pain. The eyelids were gently separated and slit-lamp examination revealed an intact globe with moderate mydriasis. There were 2+ inflammatory cells and fibrin in the anterior chamber of the right eye and corneal erosion in the left eye. Ophthalmoscopy revealed commotio retinae, and intraretinal hemorrhage at the superior quadrant with mild vitreous hemorrhage in the right eye, but no sign of a rupture of the globe.

**Figure 2:**
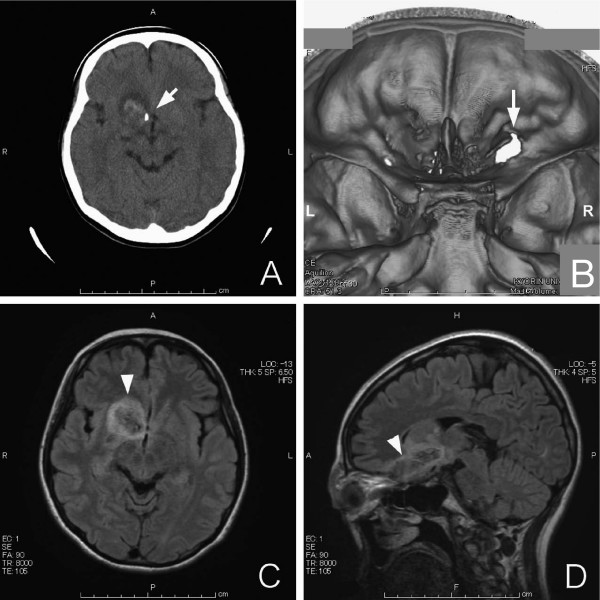
**Computerized tomographic and magnetic resonance images of the patient's brain**. A) This computed tomography (CT) scan shows incarcerated orbital bone fragment (arrow) and brain contusion. B) This three-dimensional CT scan shows a window defect caused by a fracture (arrow) of the superior orbital wall. C) This magnetic resonance image (MRI) shows the brain edema in greater detail (arrowhead). D) This MRI sagittal section shows where the umbrella tip had penetrated through the periorbital puncture (arrowhead).

Although her lid wound was small the woman had severe headache, and CT was performed on the orbit and brain to determine the extent of the injury. Unexpectedly, the images revealed irregular signals between the right orbit and the third ventricle. The signals were interpreted as brain edema, and the high-density area, which was isodense with bone and located anteriorly to the third ventricle, represented orbital bone fragments; no other foreign body was detected on cross-sectional CT scanning (Figure 2a). On three-dimensional CT scanning a window defect became apparent in the superior orbital wall (Figure 2b). MRI on the same day demonstrated edema of the head of the caudate nucleus and putamen, and the edema extended to the hypothalamus, where the bone fragments had penetrated into the brain cavity through the inferior part of the frontal lobe (Figures 2c, d). We assumed that her headaches were caused by the leakage of cerebrospinal fluid, and she was transferred to the Neurosurgery Department.

The patient was given intravenous hyperosmotic solutions to reduce the brain edema and antibiotics to prevent bacterial meningitis. Her systemic condition improved, and the ocular pain and headaches disappeared within a week. However, several neurological tests showed that her memory was altered, indicating minor brain damage.

**Figure 3:**
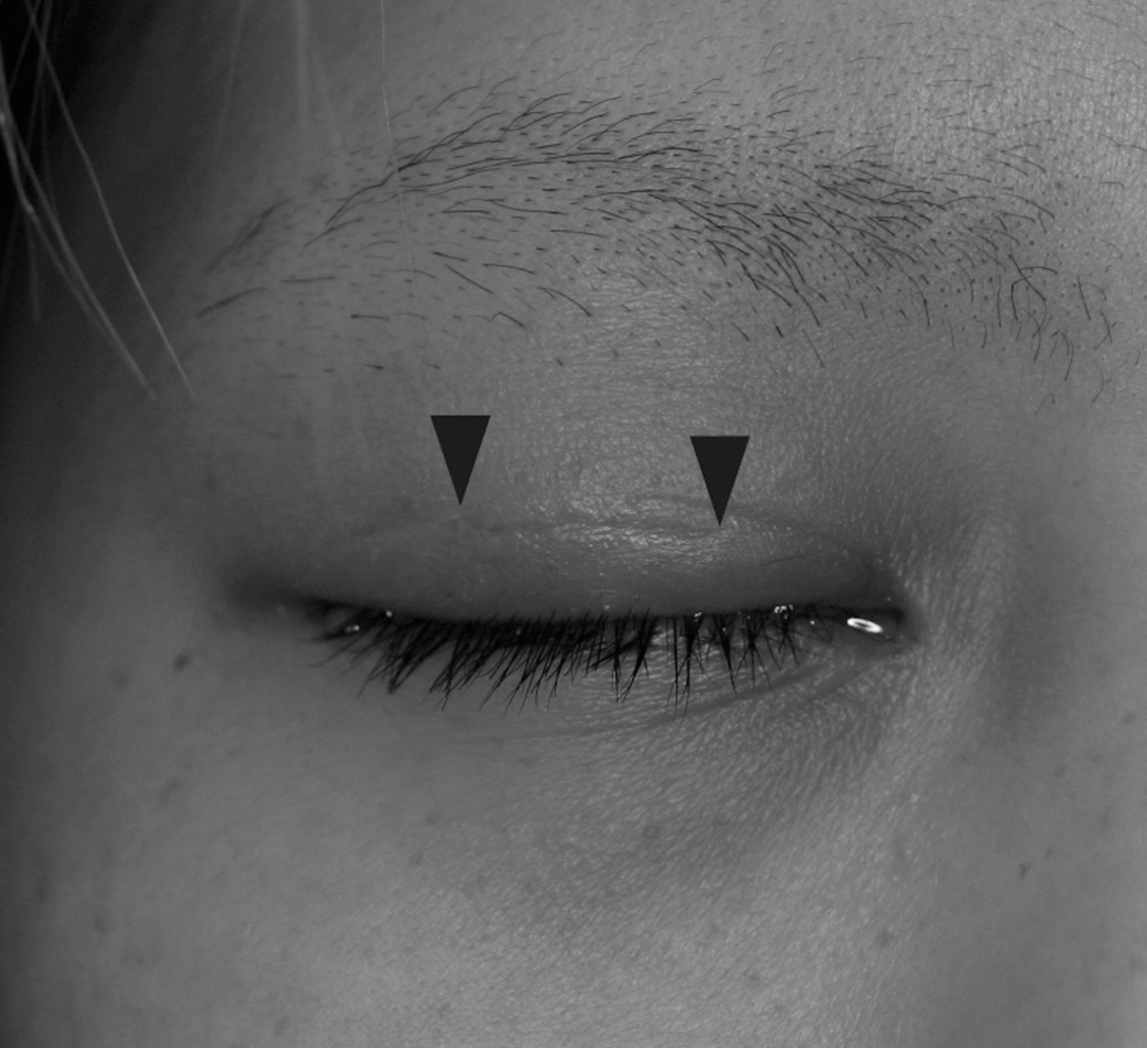
**Photograph of right eye lid**. Photograph of the patient's right eye lid three months after the injury. The width of the perforating injury is approximately 2 cm (arrowheads).

After the vitreous hemorrhage had cleared, a retinal break was found superiorly where the intraretinal hemorrhage had been located, and the break was treated by laser photocoagulation. The retina remained attached in the right eye three months later, with full ocular movement and a minor scar of width approximately 2 cm at the superior eyelid (Figure 3). Her vision improved to 20/50 in her right eye and 20/20 in her left. Decreased vision in her right eye was caused by subretinal fibrosis due to the choroidal rupture and subretinal hemorrhage caused by the blunt trauma.

## Discussion

This case demonstrates that examination of a superficial injury and descriptions of an accident by a patient do not necessarily indicate the extent of the injury. The woman described in this case report had a small penetrating injury of the eyelid and commotio retinae. However, CT and MRI of the orbit and brain showed that the umbrella tip had probably penetrated the eyelid, and the force was great enough to damage the eye and rupture the posterior orbit wall. Bone fragments were detected in the midbrain, and they were probably the cause of the edema. These findings indicated that the injury was extensive. The degree and extent of the perforating eyelid injury was overlooked by two local physicians, who did not perform brain scans.

Most orbitocranial injuries in the literature were identified in younger children who had accidentally fallen onto an object. The degree of injury was difficult to assess because of the inability of young children to describe the injury and their clinical symptoms did not always reflect the extent of damage [1-3,6]. MRI examination is recommended if a wooden object is suspected to have penetrated the brain cavity, because a wooden object can appear on a CT scan as a lucent body with nearly the same density as air or fat. Thus, a wooden object cannot necessarily be distinguished from orbital adipose tissue [2,5]. This case report demonstrates that an MRI examination is important in evaluating the extent of brain injury.

## Conclusions

Cases such as that presented here are rare. However, when a patient has symptoms that are not consistent with the observed injury (for instance, severe headache) one must maintain a high index of suspicion for severe orbitocranial injury. We recommend that MRI be performed on the orbit and brain in such a setting.

## Consent

Written informed consent was obtained from the patient for publication of this case report and any accompanying images. A copy of the written consent is available for review by the Editor-in-Chief of this journal.

## Competing interests

The authors declare that they have no competing interests.

## Authors' contributions

EN evaluated the patient. MI performed vitreous surgery. IK reviewed the manuscript. TN reviewed the manuscript. All authors read and approved the final manuscript.
